# Evolution is more repeatable in the introduction than range expansion phase of colonization

**DOI:** 10.1093/evlett/qrad063

**Published:** 2023-12-29

**Authors:** Silas Tittes, Christopher Weiss-Lehman, Nolan C Kane, Ruth A Hufbauer, Nancy C Emery, Brett A Melbourne

**Affiliations:** Institute of Ecology and Evolution, University of Oregon, Eugene, OR, United States; Department of Botany, University of Wyoming, Laramie, WY, United States; Department of Ecology and Evolutionary Biology, University of Colorado, Boulder, CO, United States; Graduate Degree Program in Ecology and Department of Agricultural Biology, Colorado State University, Fort Collins, CO, United States; Department of Ecology and Evolutionary Biology, University of Colorado, Boulder, CO, United States; Department of Ecology and Evolutionary Biology, University of Colorado, Boulder, CO, United States

**Keywords:** adaptation, experimental evolution, genetic drift, natural selection

## Abstract

How repeatable is evolution at genomic and phenotypic scales? We studied the repeatability of evolution during 8 generations of colonization using replicated microcosm experiments with the red flour beetle, *Tribolium castaneum*. Based on the patterns of shared allele frequency changes that occurred in populations from the same generation or experimental location, we found adaptive evolution to be more repeatable in the introduction and establishment phases of colonization than in the spread phase, when populations expand their range. Lastly, by studying changes in allele frequencies at conserved loci, we found evidence for the theoretical prediction that range expansion reduces the efficiency of selection to purge deleterious alleles. Overall, our results increase our understanding of adaptive evolution during colonization, demonstrating that evolution can be highly repeatable while also showing that stochasticity still plays an important role.

## Introduction

The current and future states of a population depend on the accumulation of past evolutionary processes it has experienced, where even minute differences can lead to divergent end products (Lenski & Travisano, 1994; Lenski et al., 1991). Given the diverse forms and functions evolution can generate (Lemire & Fothergill, 2006), repeated outcomes have historically been deemed improbable (Gould, 1990). Nonetheless, instances of convergent adaptive evolution in response to similar selective pressures are ubiquitous in nature (Colosimo et al., 2005; Losos et al., 1998; Nachman et al., 2003; Reid et al., 2000).

The degree to which evolutionary outcomes are repeatable depends on the spatial and temporal scales in question. Instances of repeated evolution are often simplified conceptually in terms of traits before and after selection; in reality, there will always be multiple selective pressures that vary over time and space (Burton et al., 2010; Endler, 1986; Gillespie, 2004), impacting how long a signature of repeatability will persist or how geographically widespread it will be (Holt & Gaines, 1992). Our ability to study these complexities is hindered by the inherent difficulty of sampling multiple populations that are experiencing sufficiently similar selective pressures throughout their evolutionary progressions. Highly replicated, controlled experiments that tease apart the effects of selection over space and time are essential to gain a more complete view of the repeatability of evolution.

Colonization is a multistep process where a population becomes a stable resident in a novel habitat. Colonization can be separated into three phases: introduction, establishment, and spread (Blackburn et al., 2011). Studying colonization experimentally can address outstanding questions about the repeatability of evolution by distinguishing the types of evolutionary processes that occur over space and time. When comparing replicate colonization events, the repeatability of evolution will depend on the consistency and type of evolutionary processes that arise and the amount of shared genetic variation among replicates (Schluter et al., 2004). As a general rule, we expect that evolution will be the most repeatable at the beginning of colonization during introduction to the novel environment because successful introduction requires adaptive responses to numerous selective pressures (Willoughby et al., 2018). Selective pressures will be strong during introduction if the environment differs substantially between native and introduced habitats. Assuming the population persists and becomes adapted to the introduced environment, establishment will follow, where the population size will increase to a high density and approach carrying capacity. We expect population density to be a particularly strong selective force during establishment (Roughgarden, 1971). As population density increases, increasing pressure for dispersal will ensue, contributing to the spread phase of colonization. Importantly, since the spread phase occurs via distinct processes (overcoming dispersal barriers vs. adapting to new conditions), it can occur simultaneously with establishment as edge populations spread outward and core populations continue to adapt (Blackburn, 2011). We expect evolution to be the least repeatable during the spread phase because of the increased role of genetic drift associated with low-population density at the edge of expanding ranges (Klopfstein et al., 2005). Notably, range expansion should reduce the efficiency of selection to purge deleterious variation (Peischl et al., 2013), further diminishing the repeatability of evolution during the spread phase.

Here we evaluate the repeatability of evolution during the colonization of red flour beetles, *Tribolium castaneum*, from a multigenerational microcosm experiment. Previous results from this same experiment revealed the evolution of population growth rate and dispersal propensity across replicated colonizing populations (Weiss-Lehman et al., 2017) and quantified the relative contributions of drift and selection during range expansion (Weiss-Lehman et al., 2019). We extend these analyses by quantifying the repeatability of spatial and temporal changes in allele frequencies consistent with positive selection during the three phases of colonization. We find that evolution during colonization is highly repeatable, but far less so during the spread phase compared to introduction and establishment. We also find evidence that conserved loci experienced increased allele frequency changes during the spread phase of colonization relative to changes during the two earlier phases, suggesting that there is a reduction in efficiency for natural selection to purge deleterious alleles during range expansion. Together, these results provide insights about the genetic basis of phenotypic evolution relevant to the stages of colonization and, more generally, for the degree that evolution during colonization is repeatable over short time scales.

## Methods

### Experimental design

**Figure 1.**
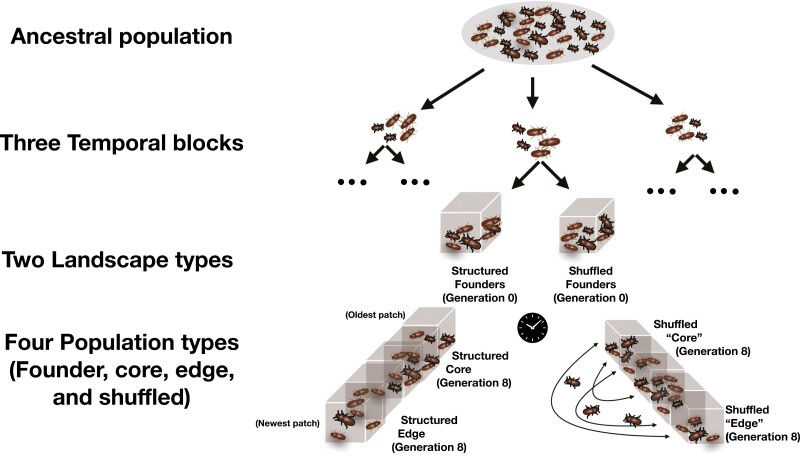
Visual summary of the experimental design for one experimental replicate. Flour beetles were sampled from an ancestral population three times, each one week apart, creating three temporal blocks. For each experimental replicate, beetles were assigned to shuffled or structured landscape types. For both landscape types, individuals mated and dispersed across patches for eight generations. The shuffled landscape randomly changed the location of individuals without modifying the patch densities. Individuals sampled from the original founding patch in generation 8 of the structured landscapes are defined as core population types, while those on the edge of the landscape form the edge population types. See *Methods* section for complete details.

Full descriptions are presented elsewhere for the experimental design (Weiss-Lehman et al., 2017) and sequence generation (Weiss-Lehman et al., 2019). We briefly reiterate them here before providing methods exclusive to this work. The experiment consisted of 60 replicated landscapes, with replicates divided evenly among three weekly blocks (referred to as “temporal blocks” throughout) for logistic feasibility(Figure 1). Each landscape provided an independent colonization starting from the same ancestral population of *T. castaneum* (originally collected from Schlegel Farm near Bloomington, IN, USA and termed the SF population) and followed colonization through introduction, establishment, and spread. Landscapes consisted of a linear series of 4 cm × 4 cm × 6 cm acrylic boxes connected via holes drilled in the sides. Each box was filled with 20 g of a homogeneous mixture of 95% wheat flour and 5% brewer’s yeast that served as food and habitat. To create each replicated landscape, we randomly sampled 20 beetles from the SF population to serve as “founders.” The SF population was kept in constant environmental conditions of 31 °C and ~60% relative humidity (RH). At the start of the experiment, the founders were moved to a new environment with constant conditions of 31 °C and ~80% RH. Both RH values were favorable growing conditions for *T. castaneum* (Howe, 1956), but the difference between the ancestral and experimental RH values created the possibility of adaptive evolution in the experimental beetles to the higher RH conditions.

To begin the experiment, the founders were placed in a patch at one end of a landscape (Figure 1). These founders were given 24 hr to reproduce and lay eggs. After this 24-hr period, the adults were sifted out, leaving the flour medium and eggs for a 33-day period allowing the eggs to develop into adults. During these first 34 days of the life cycle, dispersal was prevented. On the last day of the life cycle, barriers to dispersal were removed for 24 hr. After the dispersal period, the barriers were replaced, and the adults in each patch were censused. Adults were then placed into fresh medium for 24 hr to start the next generation. Subsequent generations followed the same procedure, with additional patches added to the landscapes each generation to ensure that populations never reached the end of a landscape. After being sifted out in the first generation, all 20 founder beetles from each landscape were stored at −80 °C for subsequent DNA extractions and analyses.

Experimental landscapes were randomly divided between two treatments, “structured” and “shuffled,” to isolate the effects of spatial spread on evolution during colonization (Figure 1). In structured populations, beetles were placed in the same patch they had dispersed to after the population census. Thus, the structured treatment maintained the spatial genetic structure formed by the colonization and expansion process. In shuffled landscapes, adults were randomly placed in patches following a population census, and the abundance of each patch determined by the census was maintained so that the shuffled treatment matched the spatial demography of the structured treatment, while removing the spatial genetic structure. Thus, landscapes in both treatments experienced the introduction and establishment phases of colonization, but only structured landscapes experienced evolution due to the spread phase, whereas the shuffled landscapes should approximate an idealized randomly mating population across the entire landscape. Landscapes in each treatment were maintained for eight generations, during which two structured replicates and one shuffled replicate were lost due to laboratory mishaps.

After eight generations, samples relevant to distinct phases of the colonization process were collected from each landscape for phenotypic assays and genomic analyses. From structured landscapes, we collected (1) a random sample of 20 beetles from the core (defined as the initial patch to which founders were first introduced), hereafter referred to as (“core populations”) and (2) the 20 beetles furthest forward at the expansion front, hereafter referred to as “edge populations” (Figure 1). Thus, the edge populations represented the spread phase of colonization while the core populations were primarily shaped by adaptation to the new laboratory conditions and informed the establishment phase. From shuffled landscapes, we collected two random samples of 20 beetles. As shuffled landscapes disrupted spatial genetic structure, these populations also represent solely the establishment phase of colonization. These samples of 20 beetles were placed in fresh media and allowed to reproduce in common garden conditions for 24 hr, after which they were stored at −80 °C for later DNA extraction and sequencing. Their offspring were then assayed to determine population growth rates across four densities and dispersal propensity across two densities (Weiss-Lehman et al., 2017). For simplicity, we will henceforth refer to these samples and their offspring as populations and refer to populations from the same location as population types (e.g., core vs. edge populations). Using this approach and comparing among founders, core, edge, and shuffled population types allowed for statistical comparison among populations representative of different phases of the colonization process (see the next three sections for further details).

### Sequence generation and processing

We pooled and extracted DNA from the 20 individuals in each population, and selected 22 structured landscapes and 15 shuffled landscapes for whole-genome sequencing (Weiss-Lehman et al., 2019). Each structured landscape provided three populations for sequencing (founders, core, and edge), while each shuffled landscape provided two (founders and a single population randomly selected from the two samples in generation 8). This yielded 96 total populations from which we constructed 96 pooled, paired-end whole-genome shotgun libraries. This resulted in 37, 22, 22, and 15 pools from the founder, core, edge, and shuffled populations, respectively, and a total of 1,920 beetles. Pools were sequenced at approximately 15X coverage. The resulting sequence reads were filtered using Trimmomatic (v0.36) (Bolger et al., 2014), and aligned to the *T. castaneum* reference genome (Tribolium Genome Sequencing Consortium, 2008) using BWA MEM (v0.7.5a-r405) (Li, 2013). Reads with alignment quality scores <20 were discarded. The RealignerTargetCreator and IndelRealigner utilities from the Genome Analysis Toolkit (v3.7-0-322 gcfedb67) (McKenna et al., 2010) were used to realign reads around indels.

We used a custom Python script (see *Data and code accessibility* for link to repository) to filter potentially spurious variation and low-quality data from our aligned sequences and generate the input data for downstream methods. Our script collated the data from all 96 pools using the SAMtools (v0.1.19-96b5f2294a) mpileup command (Li et al., 2009). We then filtered for biallelic single-nucleotide loci, discarding bases with Phred scores <20. We excluded loci with a major allele frequency >0.98 to remove possible errors and leave only well-supported allelic variation. Based on the empirical distribution of sequencing depths per locus, we chose loci with coverage between 3 and 30 alleles per pool to focus on loci likely to be single-copy. This resulted in 881,719 single-copy, well-supported polymorphic loci that were used in subsequent analyses.

### Assessing population structure

To assess the population structure that developed over the course of the experiment, we computed principal components using the prcomp function in R (R Core Team, 2019) with unit-scaled and mean-centered allele frequencies at 10K randomly subsampled polymorphic loci (results were insensitive to changing the number of loci and when repeating the procedure using different seed values). We quantified the amount of variation explained by each of the axes and visually assessed population structure based on the clustering of population types and landscapes along the first two axes.

### Identifying genomic signatures of evolution

**Figure 2.**
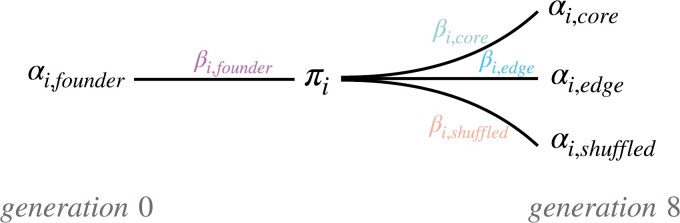
Conceptual diagram of how population allele frequencies at the *i*th locus were modeled over time and space in the experiment. The estimated departure from the grand mean allele frequency, $\pi_{i}$, was quantified as $\beta_{i}$, determining the allele frequencies, $\alpha_{i}$, for each of the four replicated experimental population types (core, edge, shuffled, and founder).

To identify candidate loci likely to be under selection at each colonization phase, we used the software Baypass (version 2.1) to identify outlier loci. Outlier loci are those with allele frequency changes that exceed what can be explained by genetic drift alone, suggesting the changes are due to positive selection. We refer to outliers as putatively selected loci throughout. We ran Baypass in Pool-Seq mode (Gautier, 2015) to infer population covariances and to construct a model to predict allele frequencies at each locus. Specifically, we used the auxiliary model (see equation 9 in Gautier, 2015), which allows a user-defined linear model based on custom covariates. Allele frequencies at each locus were modeled using a design matrix consisting exclusively of binary indicator variables for each population type (founder, core, edge, and shuffled). Hence, the model coefficients were estimates of each population type’s departure from the grand mean reference allele frequency at each locus. Our data differed from the usual application of the Baypass model in that our samples were not taken from a single time point with an unobserved ancestor. Instead, the founder populations were the observed ancestors of all generation 8 descendants (Figure 2, Table 1). As such, our estimated grand mean allele frequency at a locus (denoted $\;\pi_{i}\;$ throughout Gautier, 2015) should not be interpreted as an estimate of the ancestral allele frequency, as originally described, but instead as an intermediate allele frequency, the value of which varies according to how much allele frequencies changed across the population types during the experiment. Because colonization was replicated under the same conditions, modeling population types in this way allowed us to differentiate consistent allele frequency changes that happened at different spatial locations—those with departures from the grand mean allele frequency exclusive to edge, core, or shuffled populations, and in time—from changes exclusive to the founders, which occurred when allele frequencies changed in the same direction across descendant populations (Figure 2, Table 1). Bayes factors (BFs) at each locus were computed for each covariate, which was used to quantify the probability of an association between the allele frequencies and each of the covariates for a given locus, relative to a model that exclusively estimates the grand mean reference allele frequency. Importantly, this method computes BFs while accounting for population covariance, which reduces false positives that can arise due to variation in the amount of divergence among the populations (Gautier, 2015). We used the default Baypass settings and priors, except those for *d*_0_*y*_*ij*_, the initial value of *δy*, which is used to model the distribution of Single Nucleotide Polymorphism (SNP) allele counts in populations, which we set to eight according to recommendations in the manual, and the Ising-beta prior, which controls the degree of spatial homogeneity of neighboring variables, which we set to 0.9 to reduce the effects of autocorrelation at linked loci. We defined putatively selected loci as loci with BFs <20 on the deciban scale, which is considered decisive evidence for an association between allele frequencies at a locus and the covariate in question (Gautier, 2015). Importantly, the Baypass auxiliary model accounts for multiple testing before the BFs are computed (Gautier, 2015). Once putatively selected loci were identified, we investigated the biological relevance and potential phenotypes they were associated with using the Ensembl Metazoa (release 41) web interface for the Variant Effect Predictor (VEP) software (McLaren et al., 2016), which we used to report where the putatively selected loci occurred in the genome with respect to functionally annotated regions.

**Table 1.** Model design for predicting allele frequencies and identifying outliers with Baypass, and our interpretations for how the model predictions relate to evolution during the three phases of colonization. The left four columns show four indicator variables corresponding to the experimental population types, and the four unique corresponding row types used in the design matrix.

Indicator variables					
Founder	Core	Edge	Shuffled	Phase	Interpretation
1	0	0	0	Introduction	Shared allele frequency changes (where allele frequencies shift in the same direction for replicates of the same population type) in eighth-generation descendants. Selection resulting from differences in the native and introduced environments.
0	1	0	0	Establishment	Shared allele frequency changes among core populations. Selection resulting from high population densities and traits related to competition.
0	0	1	0	Spread	Shared allele frequency changes among edge populations. Selection resulting in traits related to dispersal ability, spatial sorting, and low population densities.
0	0	0	1	Establishment	Shared allele frequency changes among shuffled populations. Selection pressures are similar to establishment, but weaker per capita due to lower population densities relative to core populations.

### Gene ontology

For putatively selected loci that fell in annotated gene regions, we tested if any biological functions were overrepresented (by convention referred to as “enriched”) among these loci using Gene Ontology (GO) via the project’s web interface (Ashburner et al., 2000; The Gene Ontology Consortium, 2019). GO terms are standardized categories to group genes by their functional effects (Harris et al., 2004), that is, they are gene function categories. We tested for enrichment, that is, GO terms occurring in our putatively selected loci more often than can be explained by chance given their overall frequency in the genome, for the “biological process” categories (as defined by the Gene Ontology Consortium), using Fisher’s exact tests with *p* values adjusted for false discovery rate (Ashburner et al., 2000) and performed the tests for enrichment separately for each population type. Given the small number of putatively selected loci in annotated genes identified in our main analysis (Table 2), we relaxed our outlier cutoff to include loci with a BF of 10 or greater to increase our chances of identifying significantly enriched gene function categories. This increased the chance of including spuriously identified loci in the enrichment tests. However, since false positives are unlikely to occur in different genes corresponding to the same gene function category by chance, this approach provided a conservative assessment for the enrichment of gene function categories.

**Table 2.** The percentage of putatively selected, conserved, and high functional impact loci that fall into different VEP consequence categories. The first six rows show categories of loci that are within protein-coding regions, and all but the last three of the remaining rows are loci within transcribed non protein-coding regions.

Category	Putatively selected	Conserved	High functional impact
**Synonymous**	1.75	0.23	0.25
**Missense**	0.88	0.28	0.83
**Stop gained**	0.00	0.03	11.58
**Stop lost**	0.00	0.01	2.26
**Stop retained**	0.00	0.01	0.00
**Start lost**	0.00	0.00	3.34
**Splice region and synonymous**	0.00	0.01	0.00
**Start lost and splice region**	0.00	0.00	0.05
**3ʹUTR**	1.03	1.33	0.64
**5ʹUTR**	0.73	0.59	0.98
**Intron**	37.72	37.14	6.18
**Splice acceptor**	0.00	0.00	4.32
**Splice donor**	0.00	0.00	14.18
**Splice region**	0.00	0.05	0.05
**Upstream**	25.92	30.42	25.66
**Downstream**	26.50	29.23	29.20
**Intergenic**	5.20	0.03	0.00

### Changes in selection efficiency during colonization

In addition to investigating positive selection as described earlier, we also tested if the repeatability of evolution during colonization was affected by changes in the efficiency of purging deleterious variation (Peischl et al., 2013). We studied variation in the efficiency of selection during colonization phases by comparing the relative changes in the distribution of allele frequencies at conserved sites among population types. To identify the highly conserved sites in the genome, we used MUMmer (version 4) (Kurtz et al., 2004) to conduct pairwise alignments between the reference genomes of *T. castaneum*, 15 other Coleopterans, and *Drosophila melanogaster* (Supplementary Table S1). We identified all maximal exact matches of 18 or more base pairs between each taxon against the *T. castaneum* genome. We will call these putatively conserved sites. We then used Bedtools intersect (version 2.28) (Quinlan & Hall, 2010) to find putatively conserved sites that were also polymorphic in our pooled sequencing data. We found 1,836 polymorphic sites that were putatively conserved. We again used VEP to determine where variants occurred relative to coding regions, with the expectation that derived variation at sites conserved across so many species should be enriched for deleterious segregating polymorphisms. If selection alone were acting to change allele frequencies during our experiment, we would expect a reduction in, and eventual loss of derived alleles at conserved sites. Therefore, we expected that the distribution of allele frequencies would be shifted further from the reference allele frequency in the edge populations compared to those in the core and shuffled populations. Such a difference could be explained by an increase in deleterious allele frequencies at the edge, or a smaller reduction in deleterious allele frequency compared to the core or shuffled populations. We used pairs of two-sided Kolmogorov–Smirnov tests (Conover & Conover, 1980) computed in R to test for differences in the distributions of changes in allele frequencies between core and edge, core and shuffled, and edge and shuffled pools. In addition to the approach used for choosing conserved sites described earlier, we conducted the same analysis using 200 sites identified as being of high functional impact by VEP.

## Results

### Overall population structure

**Figure 3.**
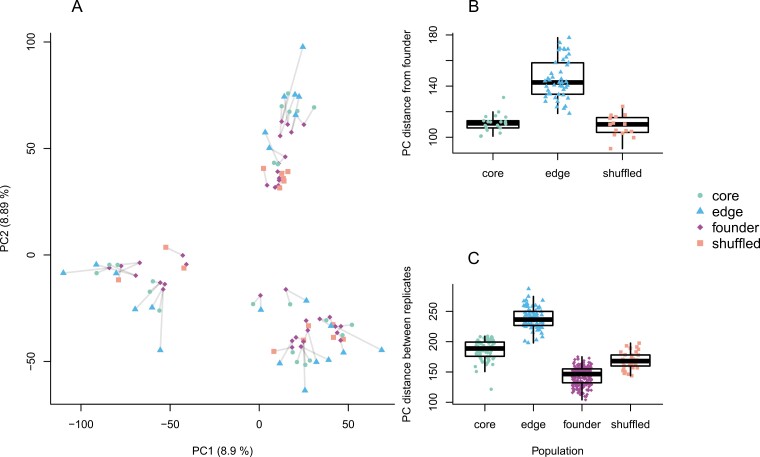
Visualization of the first two principal components of the allele frequencies shows the similarity in allele frequencies among experimental population types and recapitulates their spatial orientations within the three temporal blocks. (A) Principal components of population allele frequencies for 10K loci chosen randomly (after filtering). Light gray lines connect founding populations to their edge and core, or shuffled, eighth-generation descendants. Compared to the core and shuffled populations, edge populations were consistently farther from their founder ancestral populations (B), and farther from one another (C), as measured by Euclidean distance in principal component space calculated within each temporal block.

The first two of the 96 principal components of the allele frequencies explained 8.89% and 8.88% of the total variation (Figure 3A). The populations clustered into three distinct groups, which matched the three temporal blocks of the experiment. Within each temporal block, edge populations tended to fall on the perimeters of each cluster formed by the temporal blocks. We quantified this tendency as the Euclidean distance in principal component space, finding the edge populations were an average of 1.25 times farther from their ancestral populations than either the shuffled or core populations (Figure 3B) indicating greater genetic change in edge populations. Edge populations were also 1.3 times farther from one another than other population types were from one another, indicating greater variation among edge populations (Figure 3C). This pattern is consistent with previous analyses from this experiment that showed a stronger reduction in the correlation of allele frequencies for edge populations (Weiss-Lehman et al., 2019).

### Genomic signatures of evolution

**Figure 4.**
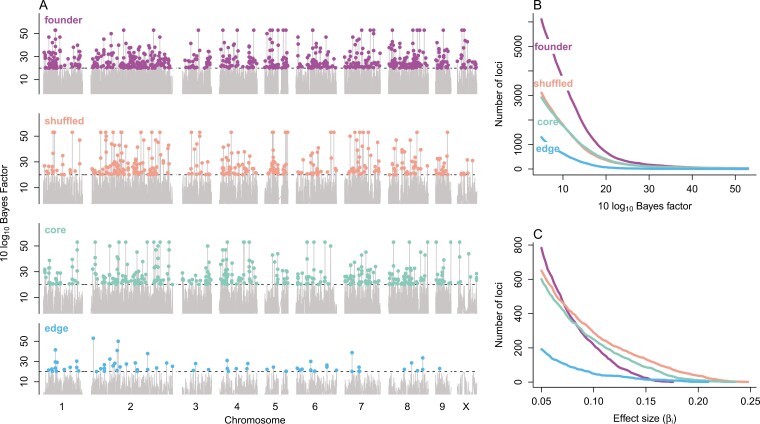
Results from the genome scan using the Baypass auxiliary model show evidence of positive selection that varied among population types. (A) Putatively selected loci (colored points) were chosen using a Bayes factor (BF) cutoff >20 deciban units, which is considered decisive evidence for an effect. The number of putatively selected loci and strength of evidence of selection varied among population types. Position on chromosomes 1–9 and X are shown. The number of loci above a given BF (B) or above a given effect size (C) reveals similar qualitative patterns about the variation among population types. Core and shuffled populations had the loci with the largest effects (C), while edge populations had the fewest putatively selected loci (B) and these had the smallest effect (C). The effect size was defined as the posterior mean of the model coefficient for each locus.

Using the Baypass auxiliary model we found a total of 1,531 putatively selected loci across all population types (founder, core, edge, and shuffled) (Figure 4A, Supplementary Table S2). Our model quantified the amount of shared allele frequency changes unique to each population type, which in turn provides evidence about changes that occurred at different phases of colonization (Table 1). The number of putatively selected loci and strength of evidence varied among population types: we found 696, 398, 366, and 71 loci with BFs >20 for the founder, core, shuffled, and edge populations, respectively. The relative number of putatively selected loci was qualitatively insensitive to varying the BF value used to define an outlier (Figure 4B), and a similar pattern was found when looking instead at the effect size of the loci (Figure 4C). Few of the putatively selected loci were found in protein coding regions. Only 1.75% and 0.88% of putatively selected loci were found at synonymous and nonsynonymous (missense) sites, respectively (Table 2).

### Gene functions

We found 4, 19, 9, and 6 of the gene function categories that were significantly enriched among putatively selected loci uniquely associated with founder, core, edge, and shuffled population types, respectively (Supplementary Table S3). Strikingly, eight of the nine significantly enriched gene function categories unique to the edge population related to the synapse (the top three were GO 0099054, 0007416, and 0099068) strongly suggesting adaptive changes in the brain occurred at the edge of expanding landscapes. The core population types showed a wider variety of categories, including categories related to actin organization (GO 0030036 and 0007015) as well as categories concerning the synapses (synaptic signaling, GO 0099536). There were four enriched categories in the founders, which reflect changes between the experimental and ancestral conditions. One intriguing category here was “cellular nitrogen compound metabolic process” (GO 0034641), which may reflect changes in the lab conditions that selected for changes in metabolism. None of the enriched GO categories in the founders involved neurological features.

### Changes in selection efficiency during colonization

**Figure 5.**
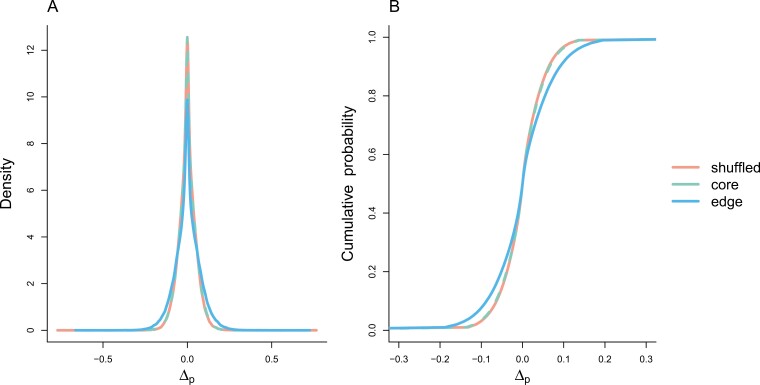
Probability density function (A) and cumulative distribution function (B) for allele frequency changes at conserved sites. The distribution for the edge population had a reduced density around zero, and an increased density toward the tails, indicating an increase in allele frequency change at conserved sites.

A total of 7,567 sites were highly conserved based on comparative analysis of reference genomes. Most of these polymorphic sites were in introns (37.14%) and upstream (30.42%) or downstream (29.23%) of coding regions (Table 2). We found evidence that the distribution of allele frequency changes at highly conserved sites was less constrained at the edge compared to core and shuffled populations (Figure 5). The distribution of changes at edge sites differed significantly from those at the core (*D* = 0.06, *p* < 2.2e-16) and from shuffled populations (*D* = 0.07 *p* < 2.2e-16), while the distribution of changes in core and shuffled populations was very similar to one another, while still being significantly different (*D* = 0.007, *p* = .002) (Figure 5). A very similar pattern was found for the 627 sites identified as having a high functional impact by VEP, though the shuffled vs. core comparison was not significant (edge vs. core: *D* = 0.06, *p* < 2.2e-16; edge vs. shuffled: *D* = 0.07, *p* < 2.2e-16; shuffled vs. core: *D* = 0.02, *p* < .058).

## Discussion

We found that adaptive evolution was common and repeatable during colonization, but that the patterns and degree of repeatability differed among the phases of colonization. Adaptive evolution was most repeatable during the introduction phase, highly repeatable during the establishment phase, and least repeatable during the expansion phase (Figure 4). Here we consider the findings for each phase.

We characterized evolution as repeatable during the introduction phase if changes in allele frequency were shared across the descendant populations compared to the founders (Table 1). Such shared changes in allele frequencies among all descendant populations could arise for several reasons. One possibility is that abiotic environmental differences in lab conditions experienced by the experimental and ancestral populations (~80% RH vs. ~60% RH respectively) could result in consistent selection on certain alleles across experimental populations. Another possibility is that traits related to density experienced different selection pressures in the ancestral populations compared to experimental conditions. Ancestral populations were maintained at constant, intermediate densities (50 individuals per patch), but experimental populations experienced substantially higher (core and shuffled populations) and lower (edge) densities. Alleles associated with optimum performance at intermediate densities would likely be selected against in all experimental populations. Regardless of the exact mechanism of selection, the large number of putatively selected loci (696 out of 1,531 total) in the introduction phase suggests that selection imposed by the experimental conditions could have involved numerous phenotypes.

To characterize evolution during the establishment phase, we separately compared allele frequencies of the shuffled or core population types to all others (Table 1). These population types represent the establishment phase because the model coefficients capture allele frequency changes that were specific to shuffled or core populations, which are the descendants that occupy the location where the founders were introduced to the new habitat. The establishment phase had roughly half the number of putatively selected loci as the introduction phase (366 in the shuffled population type and 398 in the core population type compared to 696 in the introduction phase, as discussed earlier). A broad range of GO categories were enriched in these populations, but the most intriguing were related to actin in core populations (Supplementary Table S3), which is a critical component of muscle tissue (Lehman & Szent-Györgyi, 1975). We speculate that the increased density and competition in the core could have selected for changes in body size, facilitated through changes in muscle development.

Comparing allele frequency changes for edge populations to all other population types is informative about evolution during the range expansion phase of colonization (Table 1). We found 71 putatively selected loci when comparing the edge to other population types, and the BFs and effect sizes for the edge outliers were consistently smaller (Figure 4). Observing fewer putatively selected loci in edge populations cannot be explained by a lack of statistical power to detect selection in edge populations, as we had the same number of replicates (22) from the edge population type as for the core. Furthermore, we observed relatively large increases in genetic distances between edge populations and their founders, and increased genetic distance among edge population replicates (Figure 3), both of which are indicative of the reduced correlation in allele frequencies in edge populations. Reductions in genome-wide correlation should make instances of shared allele frequency changes caused by selection at a locus easier to detect with the models employed. Instead, finding fewer outliers of smaller effect is consistent with the effects of range expansion, where predictable shifts in allele frequency driven by selection will be less common. Nonetheless, we found many enriched GO categories for the edge populations (Supplementary Table S3), many which related to neurological features, such as synapse assembly (GO:0007416). Previous research on this experiment demonstrated that dispersal tendency increased in the structured population relative to the shuffled (Weiss-Lehman et al., 2017). Finding so many GO categories related to neurological features for edge populations suggests that selection acted to change dispersal behavior. This is consistent with research in cane toads, which found the evolution of straight-line dispersal behavior increased predictably with invasion phase (Brown et al., 2014), showing the ability of selection to act on behavioral traits related to increasing the pace of range expansion.

Most outliers, regardless of the population type they were identified in, generally occurred outside of annotated and coding regions. We suspect these outliers are not causally related to phenotypes under selection but are physically linked to the causal variants, many of which will be structural variants, including INDELS, changes in repeat sequences, and copy number variants that go undetected using single nucleotide polymorphism data (Cooper & Shendure, 2011). However, of the 21 nonsynonymous outlier loci found, three occurred in genes, none were linked to previous studies on Ensemble, and many did not have names. While many of these nonsynonymous outliers may be functionally important rather than just linked to causal variants, more investigation and alternative data types would be required to make any confident claims. Specifically, the ability to differentiate among individual haplotypes and potential causal variants therein would require high-depth, long-read sequencing of individuals rather than the pooled approach used here.

In addition to edge populations having fewer putatively selected loci, they also had increased allele frequency changes at loci identified as conserved over longer periods of evolutionary time (Figure 5). This finding is predicted by theoretical models where a reduced population density increases the role of genetic drift (Slatkin & Excoffier, 2012), thus reducing the efficiency of selection to purge deleterious alleles (Peischl et al., 2013). While theoretically expected, to our knowledge, this pattern has not been previously demonstrated under controlled experimental conditions.

We recognize there are several limitations in extending our results to other organisms and timescales. The entirety of our experiment took place over eight generations, starting from a population with substantial allelic variation. This is a miniscule scale relative to much of evolutionary history. As such, there was little time for new mutations to contribute to the putatively adaptive changes we observed, with selection mainly operating on standing variation. We are limited in what the experiment can tell us about repeatability of evolution over longer time scales, as well as in other species. Nonetheless, the experiment demonstrates the ability of well-replicated microcosm experiments to provide insight into the potential outcomes of adaptive evolution, and the degree with which these outcomes are repeatable.

Using replicated colonization trials in a microcosm experiment, this study showed that adaptive evolution is most repeatable at the beginning of the colonization process during the introduction phase, highly repeatable during establishment, and least repeatable during the expansion phase. This pattern suggests that the repeatability of evolution changes over time and space during colonization. Lastly, the experiment confirmed a long-held expectation that reduced efficiency of selection during range expansion can increase the frequency of deleterious alleles.

## Supplementary Material

qrad063_suppl_Supplementary_Tables_S1-S3

## Data Availability

All sequencing data were made available in previous publications. The full list of NCBI Sequence Read Archive accessions can be found at https://dx.doi.org/10.6084/m9.figshare.c.4440284. Files and code to reproduce all figures and tables are available at https://github.com/silastittes/tribolium_pipeline and Dryad at https://doi.org/10.5061/dryad.0k6djhb6t.
