# Supplementary material for: Evolution is more repeatable in the introduction than range expansion phase of colonization
Source: Evol Lett. 2023 Dec 29;8(3):351–60. doi: 10.1093/evlett/qrad063 (PMC11134456; doi:10.1093/evlett/qrad063)
Supplement: qrad063_suppl_Supplementary_Tables_S1-S3 [file qrad063_suppl_supplementary_tables_s1-s3.zip › qrad063/Tribolium_paper2_EvoLetters_accepted_FINAL_Suppmat.pdf]

Table S1 | *Drosophila* and C*oleoptera* reference genomes and their NCBI accession numbers.

**Species**	**NCBI Accession**
Drosophila melanogaster	GCA_000001215.4
Priacma serrata	GCA_000281835.1
Hypothenemus hampei	GCA_001012855.1
Oryctes borbonicus	GCA_001443705.1
Pogonus chalceus	GCA_002278615.1
Sitophilus oryzae	GCA_002938485.1
Diabrotica virgifera	GCA_003013835.2
Aleochara bilineata	GCA_003054995.1
Harmonia axyridis	GCA_003402655.1
Dendroctonus ponderosae	GCA_000355655.1
Anoplophora glabripennis	GCA_000390285.2
Leptinotarsa decemlineata	GCA_000500325.2
Onthophagus taurus	GCA_000648695.2
Agrilus planipennis	GCA_000699045.2
Nicrophorus vespilloides	GCA_001412225.1
Aethina tumida	GCA_001937115.1

Table S2 | Complete list of putatively-selected loci and their VEP categories. The first five columns (COVARIABLE, MRK, M_Beta, SD_Beta,M_Delta, BF.dB.) are output by Baypass auxiliary model, followed by the genomic location and allele information, allele frequency (estimated with Baypass), and VEP category information.

Table S3 | Complete list of significantly enrichment Gene Ontology categories for each population type, sorted by false discovery rate adjusted p values (mean q value) averaged over the putative-selected loci that were present for each population and Gene Ontology category pair. The last column (nc) lists the number of putatively-selected loci that were included in the average q value.
